# Maternal Low-Fat Diet Programs the Hepatic Epigenome despite Exposure to an Obesogenic Postnatal Diet

**DOI:** 10.3390/nu11092075

**Published:** 2019-09-03

**Authors:** Laura Moody, Justin Shao, Hong Chen, Yuan-Xiang Pan

**Affiliations:** 1 Division of Nutritional Sciences, University of Illinois at Urbana-Champaign, Urbana, IL 61801, USA; 2 Department of Food Science and Human Nutrition, University of Illinois at Urbana-Champaign, Exeter High School, 1 Blue Hawk Drive, Exeter, NH 03833, USA; 3 Department of Food Science and Human Nutrition, Division of Nutritional Sciences, University of Illinois at Urbana-Champaign, Urbana, IL 61801, USA; 4 Department of Food Science and Human Nutrition, Division of Nutritional Sciences, and Illinois Informatics Institute, University of Illinois at Urbana-Champaign, Urbana, IL 61801, USA

**Keywords:** DO Had, developmental programming, early life nutrition, gene-environment interactions, hepatic methylome

## Abstract

Obesity and metabolic disease present a danger to long-term health outcomes. It has been hypothesized that epigenetic marks established during early life might program individuals and have either beneficial or harmful consequences later in life. In the present study, we examined whether maternal diet alters DNA methylation and whether such modifications persist after an obesogenic postnatal dietary challenge. During gestation and lactation, male Sprague-Dawley rats were exposed to either a high-fat diet (HF; *n* = 10) or low-fat diet (LF; *n* = 10). After weaning, all animals were fed a HF diet for an additional nine weeks. There were no differences observed in food intake or body weight between groups. Hepatic DNA methylation was quantified using both methylated DNA immunoprecipitation sequencing (MeDIP-seq) and methylation-sensitive restriction enzyme sequencing (MRE-seq). Overall, 1419 differentially methylated regions (DMRs) were identified. DMRs tended to be located in CpG shores and were enriched for genes involved in metabolism and cancer. Gene expression was measured for 31 genes in these pathways. *Map3k5* and *Igf1r* were confirmed to be differentially expressed. Finally, we attempted to quantify the functional relevance of intergenic DMRs. Using chromatin contact data, we saw that conserved DMRs were topologically associated with metabolism genes, which were associated with differential expression of *Adh5*, *Enox1*, and *Pik3c3*. We show that although maternal dietary fat is unable to reverse offspring weight gain in response to a postnatal obesogenic diet, early life diet does program the hepatic methylome. Epigenetic alterations occur primarily in metabolic and cancer pathways and are associated with altered gene expression, but it is unclear whether they bear consequence later in life.

## 1. Introduction

Obesogenic diets are associated with a host of chronic diseases. Calorie-dense diets, including those that are high in fat and sucrose, have been shown to blunt insulin sensitivity [1,2], increase the risk for cardiovascular disease [3,4], and increase the incidence of certain types of cancer [5,6]. While consumption of a high-fat (HF) diet is known to produce undesirable outcomes, it has been suggested that maternal diet may play an important role in preventing the negative consequences of poor postnatal diet. For instance, compared to control-fed mice, mice fed an obesogenic diet after weaning had larger adipocytes, higher fasting glucose and insulin levels, and reduced expression of insulin signaling proteins [7,8]. However, a maternal low-fat (LF) diet significantly reduced adipocyte size, lowered fasting glucose and insulin, and ameliorated the protein expression changes. Similarly, an obesogenic postnatal diet only resulted in cardiac hypertrophy and elevated fibrosis if it was preceded by an obesogenic prenatal diet, but not if it was preceded by a prenatal LF diet [9].

It has been hypothesized that perinatal nutrition acts via epigenetic mechanisms to mediate long-term health outcomes. Early life is marked by a highly dynamic epigenetic state. In particular, DNA methylation that is established during gestation is thought to persist into adulthood. Previously, maternal intake of micronutrients such as folate and choline, as well as protein restriction has been associated with altered DNA methylation in adult offspring [10,11,12]. Macronutrient consumption, particularly a HF diet, has been shown to produce methylation differences in energy homeostasis genes, peroxisome proliferator-activated receptor α (*Ppara*) [13], inflammatory genes, toll-like receptors 1 and 2 (*Tlr1* and *Tlr2*) [14], and the hepatic cell cycle inhibitor, cyclin-dependent kinase inhibitor (*Cdkn1a*) [15].

In this experiment, we examined the role of maternal diet on epigenetic programming. Pregnant Sprague-Dawley rats were fed either a HF or a LF diet during gestation and lactation. After weaning, male pups from both groups were fed a HF diet until 12 weeks of age. DNA methylation was measured in hepatic tissue using complementary methylated DNA immunoprecipitation sequencing (MeDIP-seq) and methylation-sensitive restriction enzyme sequencing (MRE-seq). Differentially methylated regions (DMRs) were characterized based on association with CpG islands and genes. Pathway analysis was performed, and gene expression was measured. Finally, we attempted to functionally interpret intergenic DMRs by examining the chromatin structure around each conserved locus.

## 2. Materials and Methods 

### 2.1. Animals and Diets

Timed-pregnant Sprague-Dawley rat dams (Charles River Laboratories, Wilmington, MA) were randomized into two groups for dietary treatment during gestation and lactation. The first group of 12 rats received a high-fat diet (HF; Research Diets, Inc.; 45% calories from fat), and the second group of 12 rats received a low-fat diet (LF; AIN93G Research Diets, Inc.; 16% calories from fat). Dams were individually housed with their pups in standard polycarbonate cages in a humidity- and temperature-controlled room on a 12-h light-dark cycle with ad libitum access to food and drinking water [16]. On postnatal day 21, male offspring (*n* = 10 rats per group from 10 different dams) were all given ad libitum access to only a HF diet until postnatal week 12. Animals were then sacrificed, and the median lobe of the liver was frozen in liquid nitrogen and stored at −70 °C. It has been shown that lobes differ in their capacity to store minerals [17], susceptibility to certain diseases [18], and transcriptomic profiles [19]. In rodents, the left lobe is developmentally distinct from the right, median, and caudate lobes. By selecting the median lobe, not only do we reduce variation between tissue samples, but we also choose a representative region that is developmentally similar to the majority of the liver. Institutional and governmental regulations regarding the ethical use of animals were followed during the study. The protocol for the ethical use of animals was approved by the Institutional Animal Care and Use Committee (IACUC protocol no. 09112).

### 2.2. Methylated DNA Immunoprecipitation (MeDIP) and Methylation-Sensitive Restriction Enzyme (MRE) Sequencing

Genomic DNA was isolated using previously published methods [20]. Animals were chosen through an extensive screening process in which gene expression and histology were measured, and the best representatives from each group were used for sequencing. Complementary MeDIP-seq and MRE-seq were then performed using previously published protocols [20]. Briefly, MeDIP utilizes antibodies against 5-methylcytidine to quantify methylated DNA sequences, while MRE-seq uses restriction enzymes that cut at unmethylated CpG sites. MeDIP-seq provides better coverage and MRE-seq offers superior resolution, so that when combined the methylome can be quantified with high accuracy [21,22]. Antibodies, restriction enzymes, DNA fragmentation, and library preparation procedures have been detailed by Li et al. [23].

### 2.3. DMR Identification

MeDIP-seq and MRE-seq data analysis were performed using the methylMnM package in R. A detailed procedure is presented by Zhang et al. [24]. In brief, the rat genome (Rn4) was partitioned into 500 bp bins, and MeDIP-seq and MRE-seq data were modeled as a function of CpG content, MRE site content, and methylation level within each bin. We used the methylMnM algorithm to test the null hypothesis that methylation level was the same between the two samples. The normalized MeDIP and MRE reads were treated as mutually independent Poisson random variables and their expected values were calculated for each sample within each bin. A test statistic and *p*-value were calculated assuming that the joint distribution of the random variables followed a multinomial distribution. Bins with a Benjamini–Hochberg false discovery rate (FDR) *p*-value <0.05 were considered significant and were called differentially methylated regions (DMRs). Further information regarding methylMnM can be found on the Bioconductor website: http://www.bioconductor.org/packages/release/bioc/html/methylMnM.html) [23,24]. 

### 2.4. Annotation and Pathway Analysis

Next, we examined the association between DMRs and CpG islands. CpG islands were defined based on three criteria: (i) sequence length greater than 200 bp, (ii) GC content greater than 50%, and (iii) an observed-to-expected CpG ratio greater than 0.6. Shores were the 2,000 bp regions upstream and downstream of each island [25]. We also annotated DMRs based on location relative to genes. DMRs were classified as either intergenic, intragenic, downstream, or in the promoter. Promoter regions were defined as the 1500 bp upstream of the transcription start site (TSS), while downstream regions were defined as the 1500 bp downstream of the transcription end site (TES) [16]. Intragenic regions included both exonic and intronic sequences of the gene body. Intergenic DMRs fell outside any gene body or 1500 bp flanking region.

Differentially methylated genes (DMGs) were annotated with Gene Ontology (GO) and Kyoto Encyclopedia of Genes and Genomes (KEGG) Pathway terms. DAVID Bioinformatics Resources version 6.7 was used to identify enriched annotation clusters and pathways with a high degree of differential methylation (http://david.abcc.ncifcrf.gov/) [26]. Functional clusters were required to have at least 2 GO and/or KEGG Pathway terms, and the majority of terms within each cluster were required to have a Benjamini–Hochberg FDR *p*-value < 0.05. Similarly, we report KEGG pathways that contained at least 2 DMGs and had a fold enrichment ≥ 1.5 (based on the proportion of a specific pathway’s genes that were DMGs).

### 2.5. Methylation Specific PCR

To validate sequencing results, methylation specific PCR (MSP) was used to quantify DNA methylation in all animals. Primer design, genomic DNA isolation, bisulfite conversion, and qPCR were performed using published methods [20]. The relative amount of methylated DNA was calculated as a ratio using the following equation: % methylated DNA = (quantity of methylated DNA)/(quantity of methylated DNA + quantity of unmethylated DNA) × 100%. All MSP primer information can be found in Table 1. 

### 2.6. Gene Expression

Total RNA was extracted using previously published methods [20]. Briefly, frozen liver tissue was ground in liquid nitrogen and the Direct-zol™ RNA MiniPrep kit (Zymo Research) was used for RNA isolation. RT-PCR was performed using the High Capacity cDNA Reverse Transcription Kit (Applied Biosystems) and incubated in a 2720 Thermal Cycler (Applied Biosystems). A serially diluted standard curve was created, and qPCR was carried out using Power SYBR^®^ Green Master Mix (Life Technologies) run in a StepOnePlus™ Real-Time PCR System. All primers were designed using Vector NTI (Life Technologies) and manufactured by Integrated DNA Technologies. Information regarding primers for gene expression is detailed in Table 2.

### 2.7. Analysis of Intergenic DMRs

In previous analyses, DMRs that are not located within close proximity of genes are often ignored. We attempted to uncover functions for these DMRs by using previously published high throughput chromatin contact data (Hi-C) [27]. Such chromatin contact maps are not publicly available for rat tissue, thus we examined only DMRs that were conserved between species using the phastCons9way track from Genome Browser. Conservation scores range from 0 to 1, where 1 denotes perfect sequence alignment and high conservation. For our analysis, we only considered DMRs that had a mean alignment score >0.5 over the 500 bp bin.

Next, the analogous region from the Rn4 genome build was identified in the Hg38 genome. We used the 3D Genome Browser to visualize Hi-C data from human liver tissue [27,28]. In particular, we examined each of the 500 bp DMRs and the 1 mb region flanking either side of the region. We then located the topologically associated domain (TAD) which contained the DMR. Genes within the TAD were considered to have contact with the nearby DMR. Those genes were selected for gene ontology and pathway analyses.

### 2.8. Statistical Analysis

For body weight, food intake, MSP, and gene expression, all pairwise comparisons between groups were made using two-tailed t-tests. For pathway analysis, significant enrichment was determined using the Benjamini–Hochberg FDR *p*-value. All statistical analysis was performed in R (version 3.1.2).

## 3. Results

### 3.1. Offspring Phenotype

**Figure 1:**
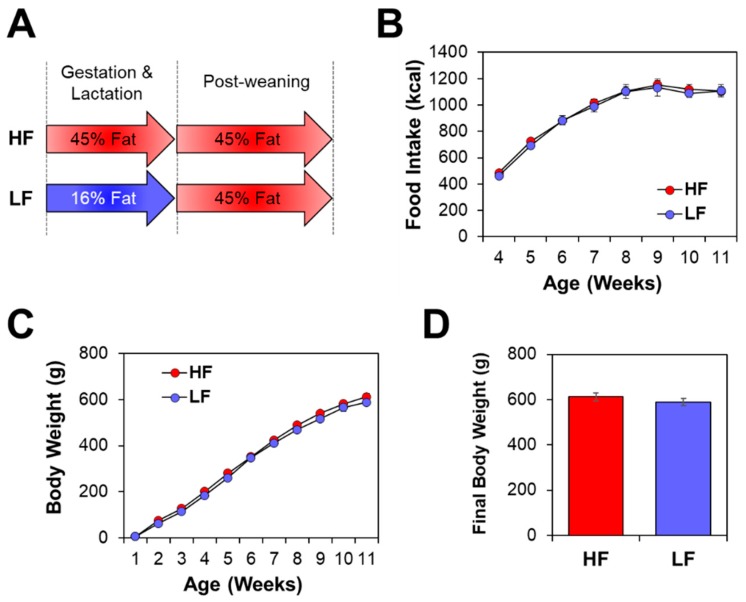
Maternal diet did not impact postnatal phenotype when followed by a high-fat (HF) diet. (**A**) Male Sprague-Dawley rats were given either a HF or low-fat (LF) diet during gestation and lactation (seven weeks). Both groups were given a HF diet after weaning (nine weeks; *n* = 10/group). (**B**) Caloric intake after weaning did not differ between the two groups. (**C**) Postnatal, and (**D**) final, body weight did not differ between groups.

Male Sprague-Dawley rats were divided into two groups and exposed to either a LF or a HF diet for seven weeks during gestation and lactation (*n* = 10/group; Figure 1A). After weaning (postnatal week three), all animals were given a HF dietary challenge that mimicked an obesogenic western diet. Animals were fed the HF diet for nine weeks and sacrificed at 12 weeks of age. Across the nine weeks of post-weaning feeding, there was no difference in food intake between the groups (Figure 1B). Additionally, body weights were consistent between groups, suggesting that maternal diet was insufficient to compensate for HF-induced postnatal weight gain (Figure 1C,D).

### 3.2. DNA Methylation

**Figure 2:**
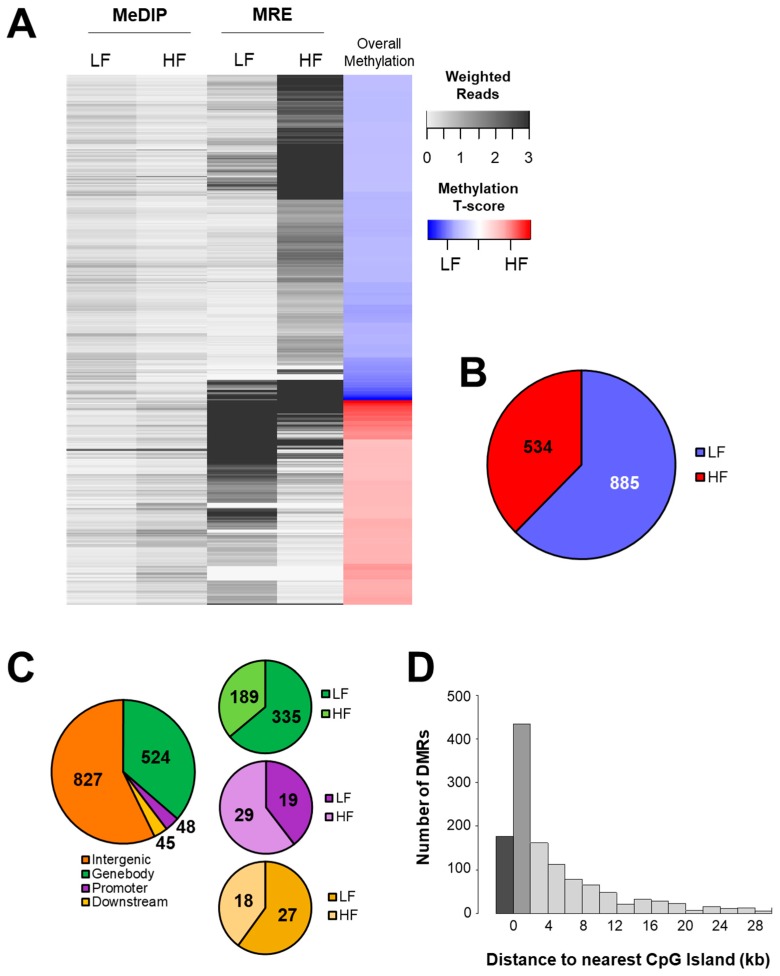
Maternal diet altered hepatic DNA methylation. (**A**) MeDIP-seq and MRE-seq were performed to quantify genome-wide DNA methylation. (**B**) Sequencing revealed 1,419 differentially methylated regions (DMRs) between groups (false discovery rate, FDR, *p*-value < 0.05), including 534 (37.6%) that were more highly methylated in the HF group and 885 (62.4%) that were more highly methylated in the LF group. (**C**) DMR position relative to genomic features, including intergenic regions, gene bodies, promoters, and downstream regions. (**D**) DMR location relative to CpG islands and shores.

Despite the lack of phenotypic differences, we hypothesized that epigenetic differences might still exist. Methylated DNA was measured using MeDIP-seq and unmethylated DNA was measured with MRE-seq. Combining the two methods, 1,419 differentially methylated regions (DMRs) were identified between groups (Figure 2A). Of these, 534 (37.6%) were more highly methylated in the HF group, while 885 (62.4%) were more highly methylated in the LF group (Figure 2B). Next, analysis of the genomic location of the DMRs revealed that 827 (58.3%) were located in intergenic regions (not within 1,500 bp of a gene), 524 (36.9%) were within the gene body (including intronic and exonic sequences), 48 (3.4%) were in a promoter (within 1,500 bp upstream of the TSS), and 45 (3.2%) were downstream of a gene (within 1500 bp downstream of the TES) (Figure 2C). We next examined DMR location relative to CpG islands and saw that more than twice as many DMRs were located within CpG shores (*n* = 435, 30.7%) than in islands themselves (*n* = 177, 12.5%, Figure 2D).

**Figure 3:**
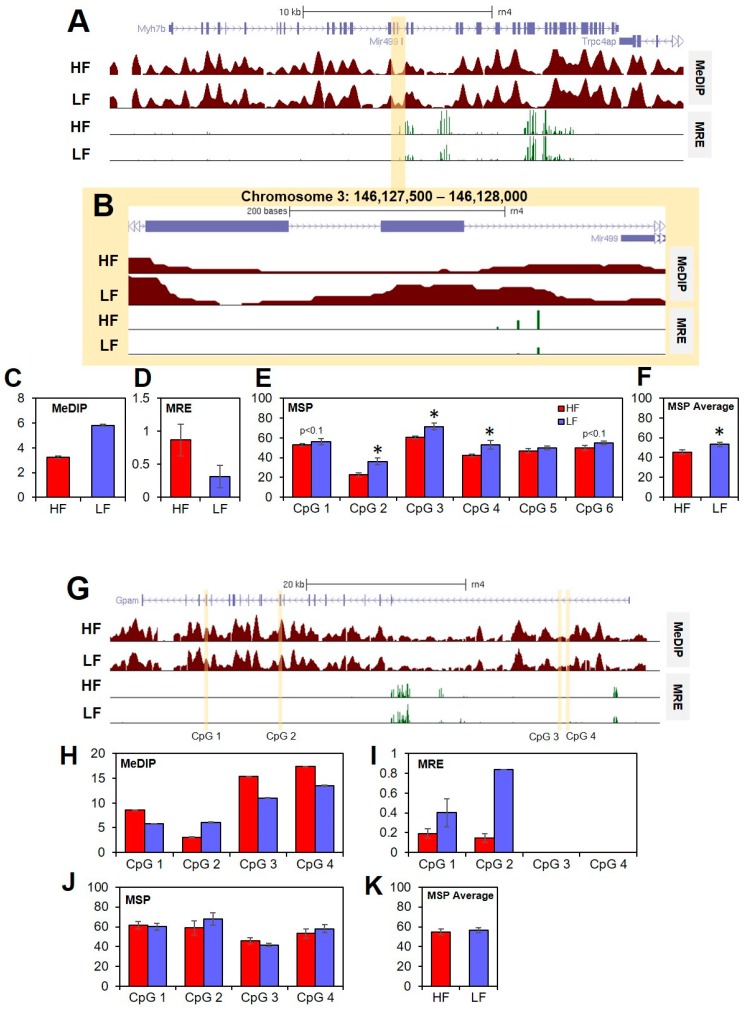
Methylation Specific PCR validates MeDIP-seq and MRE-seq findings. (**A**) Analysis of MeDIP-seq and MRE-seq identified a significant DMR within the gene body of the *Myh7b* gene (FDR *p*-value < 0.05). (**B**) The DMR spanned exon 17, intron 17/18, exon 18, and part of intron 18/19. (**C**) MeDIP-seq and (**D**) MRE-seq values are given as average reads ± standard error of the mean (SEM). (**E**) MSP was used to measure DNA methylation at six individual CpG sites within the DMR. (**F**) MSP quantities were averaged over the six sites. MSP values are reported as % DNA methylation ± SEM (* *p* < 0.05). (**G**) Among regions that were not significantly differentially methylated, four CpGs were chosen in the Gpam gene body as negative controls. (**H**) MeDIP-seq and (**I**) MRE-seq values are given as average reads ± SEM. (**J**) MSP was used to measure DNA methylation at the four CpG sites and (**K**) methylation was averaged over the four sites. MSP values are reported as % DNA methylation ± SEM (* *p* < 0.05).

DNA methylation was validated using MSP. First, we tested one DMR located within the *Myh7b* gene that was identified to be more highly methylated in the LF group by MeDIP-seq and MRE-seq (Figure 3A–D). MSP was performed on six CpG sites within the DMR. Although one site was not differentially methylated, two of the sites were higher but not significantly hypermethylated in the LF group (*p* < 0.1) and three were significantly more methylated in the LF group (*p* < 0.05, Figure 3E). Furthermore, the average DNA methylation across all six sites was significantly higher in the LF group (*p* = 0.0089, Figure 3F). Negative control was also performed on four CpG sites within the *Gpam* gene (Figure 3G). Four CpGs that were not computationally identified by MeDIP and MRE analysis were examined with MSP (Figure 3H,I). Neither the individual sites nor the average over the region showed differential methylation (Figure 3J,K). Findings here and in our previous work demonstrate the validity of combined MeDIP-seq and MRE-seq analysis [16,20].

**Figure 4:**
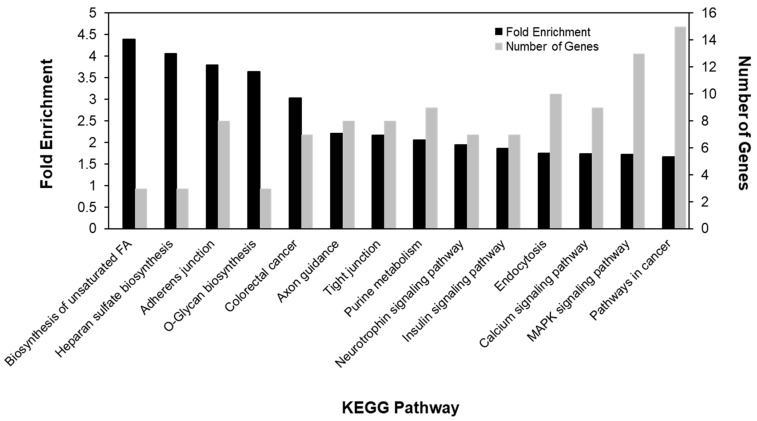
Differential methylation was most enriched in metabolic and cancer-related pathways.

In order to understand the functional relevance of the gene-associated DMRs, gene ontology (GO) and KEGG pathway analyses were performed. We found that DMGs tended to cluster in processes involved in ion binding, cell morphogenesis, ion channels, and neuronal development (Table 3). Additionally, we found that the biosynthesis of unsaturated fatty acids pathway was most highly enriched for differential methylation, while pathways in cancer contained the greatest number of differentially methylated genes (Figure 4). Interestingly, we found that other metabolic and cancer pathways were also enriched, including the insulin signaling, colorectal cancer, and mitogen-activated protein kinase (MAPK) signaling pathways.

### 3.3. Gene Expression

**Figure 5:**
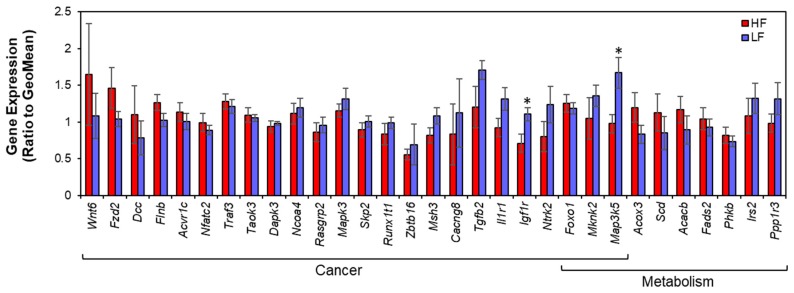
Gene expression in cancer and metabolism genes. Gene expression values are normalized to the geometric mean of *Actb*, *Gapdh*, and *Rpl7a*. * *p* < 0.05.

Given the enrichment of differential methylation in cancer and metabolic genes, qPCR was utilized to measure gene expression of 31 differentially methylated genes in those pathways. Details regarding the genomic location and methylation levels of the gene-associated DMRs can be found in Table 4. We found two differentially expressed genes, including *Map3k5* and *Igf1r* (*p* = 0.030 and *p* = 0.026, respectively; Figure 5). Both *Igf1r* and *Map3k5* were related to cancer and metabolism. Both *Map3k5* and *Igf1r* were more highly expressed in the LF group compared to the HF group. 

### 3.4. Intergenic CpGs

**Figure 6:**
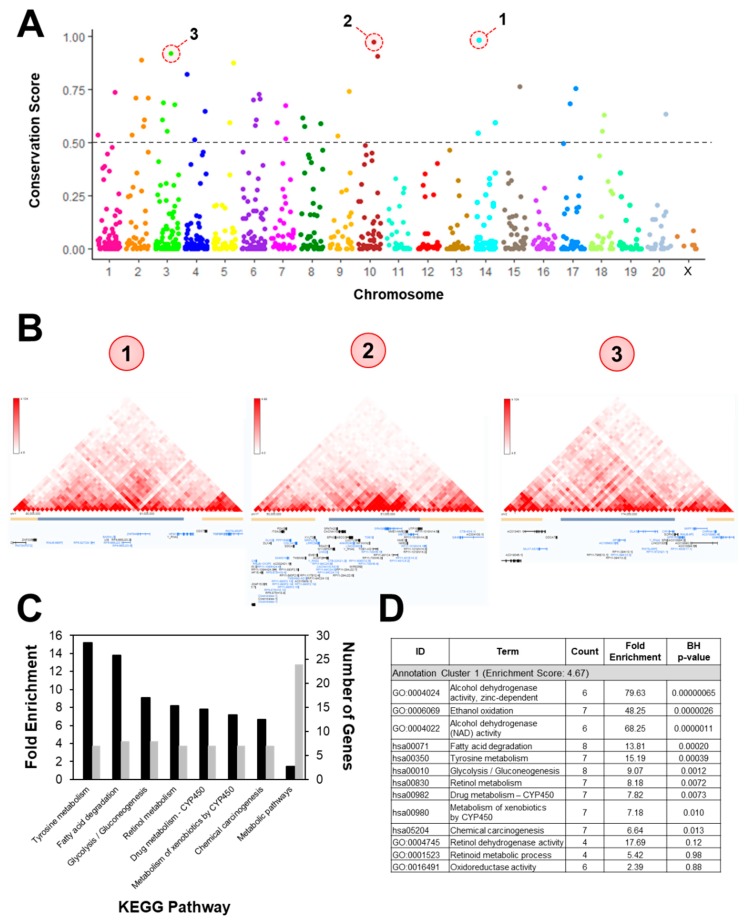
Intergenic DMRs in conserved genomic regions were associated with metabolic genes through chromatin contacts. (**A**) The conservation score was calculated for every intergenic DMR and only those with scores >0.5 were considered for further analysis (*n* = 42). (**B**) Regions from the Rn4 genome build were aligned with the Hg38 genome and queried for chromatin contacts. As an example, contact maps are shown for the three DMRs with the highest conservation scores. Genes located within the DMR-associated topologically associated domain (TAD; gray bars) were used for functional analysis. (**C**) KEGG Pathway analysis and (**D**) functional clustering showed enrichment for metabolic processes.

Finally, we attempted to understand the impact of DNA methylation in regions that were not associated with particular genes. The majority of the identified DMRs were located in intergenic regions. Previously, such CpGs have been ignored. We hypothesized that even DMRs without obvious gene contact would impact expression via altering chromatic confirmation. Three-dimensional DNA dynamics have been studied in the human genome using chromatic capture techniques; however, these methods have not been performed in rat samples. Thus, we were only able to address the 42 CpG sites that had a conservation score >0.5 (Figure 6A). From the Rn4 genome build, the analogous region was identified in the Hg38 genome. The DMR and the 1 mb region flanking either side were queried for topologically associated domains (TADs) using Hi-C data from liver tissue (Figure 6B) [27]. KEGG Pathway and Gene Ontology analyses were performed on all genes that fell within a TAD containing a DMR (Figure 6C,D). Interestingly, these genes also had functions in metabolism, replicating the findings in the gene-associated DMRs.

**Figure 7:**
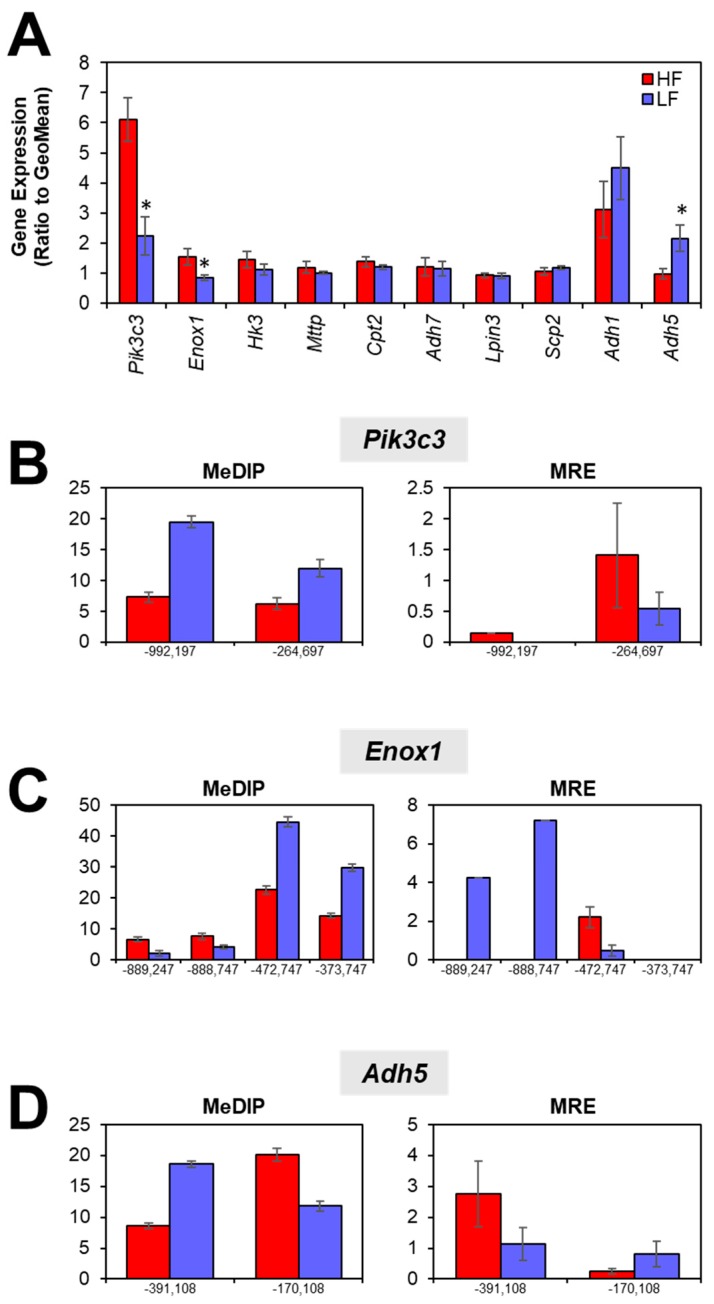
Expression of metabolic genes associated with intergenic DMRs. (**A**) Three genes were differentially expressed, including *Pik3c3*, *Enox1*, and *Adh5*. DMRs within 1 mb upstream and downstream of the gene body are reported for (**B**) *Pik3c3*, (**C**) *Enox1*, and (**D**) *Adh5*. MeDIP-seq and MRE-seq values are presented as average reads ± SEM.

We then measured the expression of ten metabolic genes associated with intergenic DMRs (Figure 7A). We found that *Pik3c3* and *Enox1* were more highly expressed in the HF group (*p* = 0.0028 and *p* = 0.025, respectively) while *Adh5* was more highly expressed in the LF group (*p* = 0.029). We then examined the DMRs within 1 mb of each gene. Two DMRs were located upstream of the *Pik3c3* TSS and were more methylated in the LF group as identified by MeDIP-seq and MRE-seq (Figure 7A). Four DMRs were located upstream of the *Enox1* TSS. The two more distant DMRs (−889,247 and −888,747 bp upstream) were hypomethylated in the LF group, while the two more proximal DMRs (−472,747 and −373,747 bp upstream) were hypermethylated in the LF group (Figure 7C). Two DMRs were located upstream of the *Adh5* TSS. MeDIP-seq and MRE-seq showed that the more distant DMR (−391,108 bp upstream) was hypermethylated in the LF group while the more proximal DMR (−170,108 bp upstream) was hypomethylated in the LF group (Figure 7D).

## 4. Discussion

In this study, we investigated the role of maternal diet in hepatic epigenomic programming. Specifically, male Sprague-Dawley rats were fed either a LF or a HF diet during gestation and lactation. After weaning, all animals were given a HF diet challenge. After nine weeks on the post-weaning diet, there was no difference in body weight between groups; however, hepatic DNA methylation was changed at 1419 loci. Closer examination of the DMRs revealed enrichment for metabolic and cancer pathways. Gene expression analysis showed that only *Map3k5* and *Igf1r* were differentially expressed. Finally, we looked at the conserved intergenic DMRs and found that they were also located nearby differentially expressed metabolic genes.

There was no difference in body weight and there were very few changes in gene expression between perinatal LF- and HF-exposed animals. Previous experiments have reported inconsistent results regarding body weight of animals that were given an obesogenic post-weaning diet preceded by different perinatal diets. After 14 weeks on a post-weaning HF diet, mice that had been exposed to a maternal HF diet had higher body weights than those exposed to a maternal LF diet [29,30]. Conversely, another study found that maternal dietary fat had no impact on body weight when male mouse offspring were fed a post-weaning HF diet for 17 weeks [31]. Finally, others have suggested that the impact of maternal diet is time dependent. In Sprague-Dawley rats, it was shown that perinatal diet did not change body weight after eight weeks of post-weaning HF feeding but did produce body weight differences after 16 weeks of post-weaning HF feeding [32]. In our case, we saw that the perinatal diet was not protective against HF-induced postnatal weight gain after nine weeks of HF feeding. Additionally, our study is limited because we did not consider any other metabolic parameters. Previous work has shown that a prenatal HF diet impacts glucose tolerance, lipid profile, and cardiovascular health [31,33,34,35]. Such physiological characteristics have also been associated with DNA methylation [36,37,38], so it is possible that the perinatal diet acts through epigenetic mechanisms to affect metabolic outcomes. 

The changes in DNA methylation but lack of body weight difference suggest that either perinatally programmed DNA methylation is slowly washed out by a new dietary challenge, or that DNA methylation programming is robust and foreshadows distinct future metabolic outcomes. Given previous findings, we hypothesize that our rats may have been sacrificed before weight differences could be observed. Because metabolic parameters such as resting metabolic rate, macronutrient oxidation, and body composition change during aging [39], it is possible that younger rats were able to compensate for perinatal perturbations. A similar principle might also explain the small number of differentially expressed genes. Younger animals might be better equipped to combat disturbances in the methylome. Conversely, it may be the case that DNA methylation established during the perinatal period is susceptible to change by post-weaning diet and thus yields no observable difference in the future metabolic outcome. Indeed, exposure to a post-weaning HF diet was shown to mitigate the epigenomic effects of the early-life diet [31]. Perinatal diet impacted 1,505 DMRs in male offspring given a post-weaning LF, but only 258 DMRs in offspring fed a post-weaning HF diet. We have also highlighted the important role of the post-weaning diet in establishing DNA methylation patterns [20]. While we previously found that a post-weaning HF diet increased body weight and impacted 3,966 DMRs, we show here that perinatal diet did not alter body weight and only affected 1419 DMRs. This suggests that post-weaning diet is a strong predictor of body weight and perhaps a more powerful determinant of DNA methylation than the perinatal diet. Future investigation should quantify the contributions of gestation, lactation, and post-weaning diet in determining DNA methylation levels in order to understand the dynamic nature of the methylome. Further work should also explore whether epigenetic alterations have the same impact on gene expression and weight gain in older individuals.

Altogether, we measured five differentially expressed genes. Two genes, *Map3k5* and *Igf1r*, contained intragenic DMRs. Previous studies suggested that the two genes respond to metabolic stressors. *Map3k5* is activated by oxidative stress and inflammation [40], and *Igf1r* participates in insulin signaling and fatty acid uptake [41,42]. Moreover, *Map3k5* has been shown to be upregulated in the adipose tissue of obese individuals while *Igf1r* is upregulated in lymphocytes of obese children [43,44]. Because a HF diet can induce oxidative stress and inflammation, it is not surprising that we observed lower expression of *Map3k5* in LF-fed animals. Furthermore, the decrease in *Igf1r* expression in the LF group might be attributed to a reduced need for IGF1-mediated fatty acid uptake. Interestingly, expression of both genes was reduced under perinatal LF conditions, but while *Igf1r* had a hypomethylated DMR, *Map3k5* contained a hypermethylated DMR. This might have been due to the position of the DMRs relative to the TSS of each gene. In *Igf1r*, the DMR was located in the first intron, where it could impact transcription in a canonical manner (i.e., increased DNA methylation resulting in decreased gene expression). The DMR associated with *Map3k5* was located much further downstream of the TSS in intron 8. Several studies have suggested that gene body methylation might have non-traditional effects on transcription, which could account for the discrepancy we observed [45,46,47]. 

Amongst the genes associated with intergenic DMRs, *Adh5*, *Enox1*, and *Pik3c3* were differentially expressed. Given their role in metabolic pathways, it is not surprising that these three genes were differentially expressed as a result of dietary treatments. The reactions catalyzed by *Adh5* and *Enox1* both involve nicotinamine adenine dinucleotide (NAD). The balance between the oxidized and reduced form of the NAD cofactor is important for driving metabolic reactions. *Pik3c3* plays a role in autophagy, which has been shown to be altered by different exposures to a HF diet [48,49,50]. We hypothesize that DNA methylation in more distant areas could be indicative of chromatin state, such that high methylation is correlated with closed chromatin [51]. This is reflected in the DNA methylation and gene expression patterns that we observed. *Pik3c3* and *Enox1* were expressed at lower levels in the LF group and the proximal DMRs were hypermethylated. Conversely, *Adh5* was highly expressed in the LF group and the proximal DMR was hypomethylated. 

This study provides insight into early life nutritional programming; however, limitations should be addressed in further experimentation. Here, we only explored dietary effects in male offspring. In previous studies, we have demonstrated sex-specific physiological and molecular changes after HF diet exposure [52,53,54]. We have also found particularly robust changes in hepatic gene expression and DNA methylation in male rats [16,20,55,56]. Although this study expands upon our prior findings in males, we cannot assume that these results would broadly apply to females. Further investigation is necessary to test whether diet-induced DNA methylation patterns are observed in both sexes. Another limitation of the current study is that epigenomic profiling was performed on whole liver tissue. While bulk tissue measurements have been previously used to uncover differences in hepatic gene expression and epigenetic profiles [57,58,59], future studies should consider isolating hepatocytes in order to better predict the functional role of differences in DNA methylation. 

Our findings represent a novel contribution to the field of nutritional epigenetics. Whereas several other studies have taken gene-targeted approaches to study DNA methylation, we interrogated the entire epigenome to uncover novel differences in DNA methylation. This allowed us to better investigate intergenic DMRs. Previous analysis has focused on gene-associated DMRs located within 1,500 bp upstream and downstream of a gene body. Indeed, certain technologies such as reduced representation bisulfite sequencing are designed to specifically identify DNA methylation differences in regions near gene promoters. Using these methods, the majority of identified DMRs have the advantage of being functionally interpretable. However, intergenic regions are often overlooked. Interestingly, we found intergenic DMRs to account for more than half of all differential methylation. Intergenic DNA methylation is thought to play an important in cellular function and disease. Intergenic DMRs can impact transcription via interaction with enhancer elements [60], and intergenic demethylation is observed in various cancers [61]. Currently, there are no bioinformatics approaches to identify specific functions for large numbers of intergenic DMRs. We hypothesized that proximal genes with significant chromatin contact were most likely to be affected by differential methylation. Although this hypothesis was supported by three differentially expressed genes, understanding the role of intergenic methylation should still be a priority in order to facilitate the development of new computational tools to annotate these regions.

## 5. Conclusions

Overall, we conclude that perinatal diet impacts hepatic DNA methylation, especially in metabolic and cancer-related pathways. On the other hand, early-life LF diet is not adequate to prevent postnatal weight gain induced by an HF diet. Although minimal, a prenatal LF diet produced changes in gene expression, including increased the expression of *Map3k5*, *Igf1r*, and *Adh5*, and decreased the expression of *Enox1* and *Pik3c3*. Our findings suggest that diet-mediated epigenetic marks established during early life persist despite a HF diet challenge during the postnatal period.

## Figures and Tables

**Table 1:** Methylation specific PCR (MSP) primers.

Gene	CpG Site	Position	Methylation	Primers (5′ → 3′)	Efficiency *
Gpam	CpG 1	Forward +52,479	U	GTAGTGGAATAAGAAGTTTTCGGAG	98.20%
		Reverse +52,587	U	ACCTTCAAATAACAATCACGCTAC	
		Forward +52,485	M	AGAGTAGTAGTGGAATAAGAAGTTTTTGG	99.10%
		Reverse +52,586	M	CACCTTCAAATAACAATCACACTAC	
	CpG 2	Forward +44,337	U	GGTGGAGAGGTATTTGTTATGGA	108.20%
		Reverse +44,416	U	CCACACAATCTACAAACTTCACAA	
		Forward +44,332	M	TTAGAGGTGGAGAGGTATTTGTTAC	101.19%
		Reverse +44,414	M	ACACAATCTACAAACTTCACGAAAA	
	CpG 3	Forward +7619	U	TGTAATTTTTTATTTTAATTTATGTGATTTTTGA	95.56%
		Reverse +7736	U	TTTCTACTTCACAATTACTAATCAACCCA	
		Forward +7633	M	TTAATTTACGTGATTTTTGATTGTTATTATTTT	98.50%
		Reverse +7736	M	CTACTTCACGATTACTAATCAACCCG	
	CpG 4	Forward +7288	U	AAGTTAAGTTGTAGTGGTTGGGTAATTG	93.47%
		Reverse +7360	U	CCCACTTATTTTAAACAACATCAAACC	
		Forward +7289	M	AGTCGTAGTGGTCGGGTAATCG	107.81%
		Reverse +7357	M	CCGCTTATTTTAAACAACATCGAA	
Myh7b	CpG 1	Forward +9089	U	TGGGTTCGTGTGGGAAATG	99.33%
		Reverse +9181	U	CCACCTCAACTCTCCCTAAACAA	
		Forward +9089	M	CGGGTTCGTGTGGGAAAC	92.99%
		Reverse +9181	M	CCGCCTCAACTCTCCCTAAA	
	CpG 2	Forward +9607	U	AGGAGTATAAATGGGAGGGTATTGATT	91.86%
		Reverse +9757	U	CATACACAACTTCCAACACCATCC	
		Forward +9621	M	CGTGTTGGAGTAGGAGGAGTATAAAC	102.11%
		Reverse +9753	M	CGCAACTTCCGACACCATC	
	CpG 3	Forward +10,000	U	TGATTTGAGGATTATGTGTATTGGATTT	99.04%
		Reverse +10,069	U	CCAATTTCTTTTTCCATTCTCCATAC	
		Forward +9995	M	CGAGGATTACGTGTATTGGATTTTAA	103.13%
		Reverse +10,065	M	TTTCTTTTTCCATTCTCCGTACAATA	
	CpG 4	Forward +11,606	U	AGGAGTGTATGTTTTTTAAGGTTTTAGATG	96.42%
		Reverse +11,701	U	AACAAAACTACTAAAAATTAAATAACTTCCCA	
		Forward +11,606	M	AGGAGTGTATGTTTTTTAAGGTTTTAGACG	98.73%
		Reverse +11,701	M	ACGAAACTACTAAAAATTAAATAACTTCCCA	
	CpG 5	Forward +12,857	U	GATTTGGATTTGTTGTTAAGGGTTTT	102.17%
		Reverse +12,924	U	AACCAACACCCACCACTACCTAA	
		Forward +12,859	M	TTCGGATTTGTCGTTAAGGGTT	106.16%
		Reverse 12,927	M	CCAACACCCACCGCTACC	
	CpG 6	Forward +13,120	U	TTATTTGGATATGGGATAAGAGAGGG	98.27%
		Reverse +13,216	U	CACCATCTAAAATAATACTACTTTCTTTCACTTAT	
		Forward +13,120	M	TTATTTGGATACGGGATAAGAGAGG	102.64%
		Reverse +13,219	M	CGTCTAAAATAATACTACTTTCTTTCGCTT	

* The qPCR amplification efficiency is calculated based on the slope of the standard curve https://www.lifetechnologies.com/us/en/home/brands/thermo-scientific/molecular-biology/molecular-biology-learning-center/molecular-biology-resource-library/thermo-scientific-web-tools/qpcr-efficiency-calculator.html. Slopes between −3.1 and −3.6 giving reaction efficiencies between 90 and 110% are typically acceptable.

**Table 2:** Gene expression primers.

Gene	Ensembl ID	Common Name	Position	Primers (5′ → 3′)	Efficiency *
Acacb	ENSRNOT00000078868.1	Acetyl-CoA Carboxylase Beta	Forward +1081	ACCCCAAACTTCCAGAGC	105.70%
			Reverse +1189	TGGGCTACAATGGTGGAG	
Acox3	ENSRNOT00000049798.3	Acyl-CoA Oxidase 3, Pristanoyl	Forward +1553	TGACTGGTTGGACTCAGA	94.56%
			Reverse +1629	TCTGATGACTCTCTCGGA	
Actb	ENSRNOT00000042459.4	Actin Beta	Forward +451	GAGACCTTCAACACCCCAGC	104.67%
			Reverse +526	CAGTGGTACGACCAGAGGCA	
Acvr1c	ENSRNOT00000059280.4	Activin A Receptor Type 1C	Forward +511	TGATTTATGATGCCACTGCC	100.24%
			Reverse +586	ATTGTCCTTGCGATGGTTCT	
Adh1	ENSRNOT00000036993.4	Alcohol Dehydrogenase 1	Forward +257	ATGAAGGAGTTGGGATAG	100.37%
			Reverse +318	ATCACCTGTTCTTACGCT	
Adh5	ENSRNOT00000016891.6	Alcohol Dehydrogenase 5	Forward +637	GTGTGTCTGATTGGATGTGG	117.98%
			Reverse +700	TGACCTTGGCAGTGTTGA	
Adh7	ENSRNOT00000015870.4	Alcohol Dehydrogenase 7	Forward +262	GAAGCAGTTGGGATTGTGGAGA	108.18%
			Reverse +328	TCACTTTGTCACCTGGTCTCACTG	
Cacng8	ENSRNOT00000078444.1	Calcium Voltage-Gated Channel Auxiliary Subunit Gamma 8	Forward +297	CTGCGTGAAGATCAACCACT	108.34%
			Reverse +395	ATAGGAAAGATGCTGGAGGC	
Cpt2	ENSRNOT00000016954.3	Carnitine Palmitoyltransferase 2	Forward +310	GACACCATGAAGAGATACCT	107.28%
			Reverse +387	ACACAACGCTTCTGTTCT	
Dapk3	ENSRNOT00000027634.4	Death Associated Protein Kinase 3	Forward +624	TTCGTCGCCCCTGAGATTGTAA	103.21%
			Reverse +685	ATGACGCCGATGCTCCACATAT	
Dcc	ENSRNOT00000064947.3	DCC Netrin 1 Receptor	Forward +1144	GTGGCTGAAAATGAGGCTGGC	100.76%
			Reverse +1208	ATGGCAGGCTTGGGGACAA	
Enox1	ENSRNOT00000074868.2	Ecto-NOX Disulfide-Thiol Exchanger 1	Forward +167	TTGAGAGCATCGCACAGTGT	93.82%
			Reverse +239	ATGCTCCCCAAACCATCA	
Fads2	ENSRNOT00000059280.4	Fatty Acid Desaturase 2	Forward +711	CGTGTTTGTCCTTGGAGAGTGGC	108.89%
			Reverse +790	CATGCTGGTGGTTGTAGGGCA	
Flnb	ENSRNOT00000066546.1	Filamin B	Forward +353	GCTGGAGAATGTGTCTGT	108.69%
			Reverse +422	ACTGTCAATGGACACGAG	
Foxo1	ENSRNOT00000018244.5	Forkhead Box O1	Forward +994	AGGATAAGGGCGACAGCAACAG	102.12%
			Reverse +1056	GGGACAGATTGTGGCGAATTG	
Fzd2	ENSRNOT00000032944.2	Frizzled Class Receptor 2	Forward +818	TTTTGCCCGTCTCTGGAT	93.13%
			Reverse +889	TAGGTGGTGACCGTGAAGAA	
Gapdh	ENSRNOT00000050443.4	Glyceraldehyde-3-Phosphate Dehydrogenase	Forward +220	CTCTACCCACGGCAAGTTCAACG	100.39%
			Reverse +311	CTCGCTCCTGGAAGATGGTGATG	
Hk3	ENSRNOT00000031935.2	Hexokinase 3	Forward +973	CCCTGGTTCCTGGTGCTCAG	119.40%
			Reverse +1050	CCAGCACCAGCCTTACCAGC	
Igfr1	ENSRNOT00000019267.6	Insulin Like Growth Factor 1 Receptor	Forward +2239	CTGAGAGGAGGCGGAGAGATG	109.39%
			Reverse +2304	TGTTCCTGCTTCGGCTGG	
Il1r1	ENSRNOT00000019673.4	Interleukin 1 Receptor Type 1	Forward +393	GGGTTCATTTGTCTCATTGTGC	101.20%
			Reverse +465	TGACCTCATTTGGATACTCCGT	
Irs2	ENSRNOT00000032918.6	Insulin Receptor Substrate 2	Forward +3373	CTTGAAGCGGCTAAGTCT	109.86%
			Reverse +3435	TGGCTGACTTGAAGGAAG	
Lpin3	ENSRNOT00000022403.5	Lipin 3	Forward +578	CCCTCATCGCAGCCTAAAGACAT	108.38%
			Reverse +657	AGGTCAGCAGATGAAAGGTTGGC	
Map3k5	ENSRNOT00000051496.6	Mitogen-Activated Protein Kinase Kinase Kinase 5	Forward +455	GTTTTTACAACGCTGACATCGC	105.77%
			Reverse +525	ATGATAAAACAGGGAAGGCTGC	
Mapk3	ENSRNOT00000087625.1	Mitogen-Activated Protein Kinase 3	Forward +618	CACTGGCTTTCTTACCGAGT	111.47%
			Reverse +696	GGTGTAGCCCTTGGAGTTAA	
Mknk2	ENSRNOT00000041106.5	MAP Kinase Interacting Serine/Threonine Kinase 2	Forward +177	TTCAGGGCTTCCACCGTTCG	107.33%
			Reverse +246	TGGGCGGGGTCTAAGGTGAA	
Msh3	ENSRNOT00000018449.7	MutS Homolog 3	Forward +166	TGTCCCCCACAGAACCAGCA	109.37%
			Reverse +229	TTCCCCAGTGACCTCTTCCTGC	
Mttp	ENSRNOT00000014631.6	Microsomal Triglyceride Transfer Protein	Forward +1042	TAGAACCTGAGAACCTGTCCAACGC	107.54%
			Reverse +1113	AAGTGCGGAGGTGCTGAATGAAG	
Ncoa4	ENSRNOT00000066062.3	Nuclear Receptor Coactivator 4	Forward +611	CCTAGTTCTTCAAGTGTCAGGC	108.75%
			Reverse +686	TGGATGCTGACTTCTGCTCT	
Nfatc2	ENSRNOT00000065615.1	Nuclear Factor Of Activated T Cells 2	Forward +1599	GGAGCCAAAGAACAACATGCGGG	100.47%
			Reverse +1674	CAGCTCGATGTCAGCGTTTCGGA	
Ntrk2	ENSRNOT00000042145.2	Neurotrophic Receptor Tyrosine Kinase 2	Forward +979	TCCTGGACAAACTCGTCA	99.47%
			Reverse +1058	GGCTTACAAGGCGTTTCT	
Pik3c3	ENSRNOT00000066816.2	Phosphatidylinositol 3-Kinase Catalytic Subunit Type 3	Forward +60	CTGTGACCTGGACATCAA	93.26%
			Reverse +119	TGTTCTCTCTTCCCTTCC	
Phkb	ENSRNOT00000049624.4	Phosphorylase Kinase Regulatory Subunit Beta	Forward +369	GCCATAAAGTGTATGAGAGGAG	106.60%
			Reverse +435	TGAACTGCTGGACCTTATCA	
Ppp1r3b	ENSRNOT00000051720.2	Protein Phosphatase 1 Regulatory Subunit 3B	Forward +838	TATGAAAGAATGGAGTTCGCCGTG	107.84%
			Reverse +909	TTTGCCTTTGTTGCTGTCCCAGTA	
Rasgrp2	ENSRNOT00000028646.6	RAS Guanyl Releasing Protein 2	Forward +490	CAAGAAGGAAACCGCAGGCAC	108.06%
			Reverse +565	TCACCTGCCGCTTCCACTTGT	
Rpl7a	ENSRNOT00000044551.4	Ribosomal Protein L7a	Forward +64	GAGGCCAAAAAGGTGGTCAATCC	105.33%
			Reverse +127	CCTGCCCAATGCCGAAGTTCT	
Runx1t1	ENSRNOT00000066191.1	RUNX1 Translocation Partner 1	Forward +796	TCCCACTGAGACCTTTTG	109.60%
			Reverse +894	CAGGGTTCTGTTTGGCTA	
Scd	ENSRNOT00000018447.5	Stearoyl-CoA Desaturase	Forward +942	TCAATCTCGGGAGAACATCCTG	109.77%
			Reverse +1013	AAGGCGTGATGGTAGTTGTGGA	
Scp2	ENSRNOT00000015420.5	Sterol Carrier Protein 2	Forward +203	GGCTATGTGTACGGTGAATCCA	105.56%
			Reverse +280	AATGATAGGGATGCCAGTCAGC	
Skp2	ENSRNOT00000089178.1	S-Phase Kinase Associated Protein 2	Forward +809	CTGGATTTTCTGAGTCTGCC	100.52%
			Reverse +882	CCAGGAGAGGTTCAGTTCAT	
Taok3	ENSRNOT00000089043.1	TAO Kinase 3	Forward +403	GCTGAAGCACCCGAACACCAT	103.35%
			Reverse +476	ACTCCATCACCAACCAAGCGG	
Tgfb2	ENSRNOT00000003313.5	Transforming Growth Factor Beta 2	Forward +1664	ACAATGCTAACTTCTGTGCTGG	91.24%
			Reverse +1735	TGAGGACTTTGGTGTGTTGTGT	
Traf3	ENSRNOT00000010906.6	TNF Receptor Associated Factor 3	Forward +870	CTCTTCTGAGGAGTGAGTTGA	108.60%
			Reverse +942	CGCTTAAAACTACAGGTGC	
Wnt6	ENSRNOT00000023439.6	Wnt Family Member 6	Forward +452	GGGGGTTCGAGAATGTCAGTTCC	103.79%
			Reverse +517	GCCTTGCTGTGACTGGAGCAGTT	
Zbtb16	ENSRNOT00000045356.3	Zinc Finger and BTB Domain Containing 16	Forward +1695	GCATTTACTGGCTCATTCAG	100.89%
			Reverse +1770	ATCTTCCTTTGAGAACTGGG	

* The qPCR amplification efficiency is calculated based on the slope of the standard curve https://www.lifetechnologies.com/us/en/home/brands/thermo-scientific/molecular-biology/molecular-biology-learning-center/molecular-biology-resource-library/thermo-scientific-web-tools/qpcr-efficiency-calculator.html. Slopes between −3.1 and −3.6 giving reaction efficiencies between 90 and 110% are typically acceptable.

**Table 3:** Gene ontology clustering.

GO Number	GO Annotation	Count	Fold Enrichment	FDR *p*-Value
Annotation Cluster 1 (Enrichment Score: 6.24)				
GO:0046872	Metal ion binding	111	1.54	2.37 × 10^−4^
GO:0043167	Ion binding	113	1.52	1.70 × 10^−4^
GO:0043169	Cation binding	111	1.52	1.61 × 10^−4^
Annotation Cluster 2 (Enrichment Score: 5.24)				
GO:0000902	Cell morphogenesis	26	3.02	0.0013
GO:0032989	Cellular component morphogenesis	26	2.73	0.0045
GO:0000904	Cell morphogenesis involved in differentiation	20	3.27	0.0046
Annotation Cluster 3 (Enrichment Score: 4.57)				
GO:0005216	Ion channel activity	24	2.83	0.0016
GO:0005261	Cation channel activity	20	3.18	0.0017
GO:0022838	Substrate specific channel activity	24	2.75	0.0018
GO:0015267	Channel activity	24	2.65	0.0028
GO:0022803	Passive transmembrane transporter activity	24	2.65	0.0028
GO:0022836	Gated channel activity	20	2.95	0.0033
Annotation Cluster 4 (Enrichment Score: 4.18)				
GO:0048667	Cell morphogenesis involved in neuron differentiation	19	3.63	0.0029
GO:0000904	Cell morphogenesis involved in differentiation	20	3.27	0.0046
GO:0007409	Axonogenesis	17	3.61	0.0056
GO:0048666	Neuron development	23	2.62	0.014
GO:0048812	Neuron projection morphogenesis	17	3.12	0.021
GO:0031175	Neuron projection development	19	2.75	0.034
GO:0048858	Cell projection morphogenesis	17	2.82	0.045
GO:0032990	Cell part morphogenesis	17	2.71	0.066
Annotation Cluster 5 (Enrichment Score: 4.08)				
GO:0022836	Gated channel activity	20	2.95	0.0033
GO:0005244	Voltage-gated ion channel activity	15	3.47	0.0062
GO:0022832	Voltage-gated channel activity	15	3.47	0.0062
Annotation Cluster 6 (Enrichment Score: 3.45)				
GO:0051960	Regulation of nervous system development	17	2.95	0.036
GO:0060284	Regulation of cell development	17	2.87	0.044
GO:0045664	Regulation of neuron differentiation	14	3.31	0.042
GO:0050767	Regulation of neurogenesis	15	2.86	0.086

**Table 4:** DMR description.

Gene	Function	Chromosome	Location	Genomic Feature	HF MeDIP	LF MeDIP	HF MRE	LF MRE	Conservation
ACVR1C	Cancer	3	40041000–40041500	Intron 3/8	2.33 (0.89)	2.58 (1.02)	1.64 (0)	0	0.0040 (0.016)
CACNG2	Cancer	7	116000000–116000500	Intron 1/3	8.27 (3.47)	12.26 (4.78)	0.74 (0.42)	0.31 (0.24)	0.77 (0.36)
CACNG8	Cancer	1	64069000–64069500	Exon 4/4	2.56 (1.15)	2.00 (0.82)	0.27 (0)	6.07 (0)	0.18 (0.34)
DAPK3	Cancer	7	10009000–10009500	Exon 7/9	12.89 (6.18)	18.09 (10.40)	1.10 (0.68)	0.14 (0)	0.38 (0.45)
DCC	Cancer	18	69043500–69044000	Intron 1/28	8.56 (3.39)	21.00 (9.64)	3.68 (1.51)	2.16 (1.46)	0.0054 (0.0087)
FLNB	Cancer	15	19052000–19052500	Intron 1/44	4.89 (2.41)	5.98 (2.59)	2.06 (2.03)	0.51 (0.58)	0.014 (0.017)
FZD2	Cancer	10	91707500–91708000	Promoter	6.27 (3.41)	1.40 (0.55)	0.27 (0.21)	0.95 (0.71)	0.061 (0.094)
IGF1R	Cancer	1	122713500–122714000	Intron 1/20	4.31 (1.20)	3.10 (1.81)	0.71 (0)	1.39 (0.99)	0.0057 (0.011)
IL1R1	Cancer	9	39589500–39590000	Intron 1/10	7.39 (4.28)	4.33 (1.62)	0.12 (0)	1.00 (0.93)	0.0053 (0.0099)
MAP3K5	Cancer	1	15307000–15307500	Intron 8/12	1.88 (0.83)	4.86 (2.15)	7.37 (0)	1.13 (0)	0.018 (0.050)
MSH3	Cancer	2	22480500–22481000	Intron 20/23	10.93 (4.44)	4.08 (2.34)	0.42 (0.42)	1.60 (0.93)	0.0050 (0.014)
NCOA4	Cancer	16	7641000–7641500	Promoter	12.59 (3.16)	5.25 (2.38)	0.27 (0.085)	1.10 (0.84)	0.0026 (0.0036)
NFATC2	Cancer	3	159682000–159682500	Intron 8/9	10.58 (5.74)	3.06 (1.24)	1.07 (0)	1.73 (2.32)	0.015 (0.0038)
NTRK2	Cancer	17	11700500–11701500	Intron 12/13	7.81 (3.75)	17.72 (8.57)	4.31 (3.07)	1.21 (1.92)	0.28 (0.41)
RASGRP2	Cancer	1	209198000–209198500	Intron 15/16	4.29 (2.91)	6.10 (4.07)	0.85 (0.14)	0.19 (0)	0.22 (0.33)
RUNX1T1	Cancer	5	28230500–28231000	Exon 1/11	2.63 (1.09)	1.00 (0)	1.70 (1.81)	8.70 (11.88)	0.31 (0.43)
SKP2	Cancer	2	58774500–58775000	Exon 8/10	8.21 (4.43)	17.98 (10.69)	0.93 (1.08)	0	0.13 (0.30)
TAOK3	Cancer	12	40571000–40571500	Intron 5/19	12.23 (7.54)	7.64 (3.88)	1.51 (2.00)	4.16 (5.87)	0.23 (0.41)
TGFB2	Cancer	13	102723000–102723500	Intron 6/7	10.45 (6.36)	4.61 (2.68)	0.24 (0)	3.47 (0.13)	0.39 (0.47)
TRAF3	Cancer	6	135924000–135924500	Promoter	5.27 (2.45)	1.50 (0.71)	0.47 (0.57)	10.45 (21.93)	0.036 (0.073)
WNT6	Cancer	9	74115500–74117500	Intron 3/3	8.30 (6.35)	15.22 (7.88)	3.40 (4.52)	3.54 (5.89)	0.26 (0.43)
ZBTB16	Cancer	8	52035000–52036000	Intron 2/6	5.24 (2.69)	7.58 (2.93)	4.00 (0.63)	0.70 (0.30)	0.45 (0.49)
FOXO1	Cancer/Metabolism	2	141195500–141196000	Intron 1/2	4.84 (2.03)	1.33 (0.58)	0.18 (0.084)	1.31 (0.27)	0.013 (0.026)
MAPK3	Cancer/Metabolism	1	185936000–185936500	Exon 2/10	7.00 (3.22)	13.88 (6.77)	8.62 (0)	2.72 (0)	0.74 (0.39)
MKNK2	Cancer/Metabolism	7	10559000–10559500	Exon 14/14	8.85 (3.70)	14.22 (6.72)	3.20 (2.54)	0.98 (0.97)	0.32 (0.42)
ACACB	Metabolism	12	43424500–43425000	Intron 30/58	4.67 (2.22)	5.63 (2.80)	9.21 (0.59)	3.01 (1.59)	0.0020 (0.0041)
ACOX3	Metabolism	14	80781000–80781500	Intron 13/19	15.48 (9.69)	3.00 (1.58)	0	0	0.0047 (0.016)
FADS2	Metabolism	1	212532500–212533000	Intron 4/10	7.83 (3.78)	12.44 (4.55)	1.67 (1.44)	0.87 (0.92)	0.27 (0.44)
IRS2	Metabolism	16	83384000–83384500	Intron 1/1	9.39 (4.22)	1.67 (0.71)	0.24 (0.17)	2.82 (0.27)	0.00077 (0.0032)
PHKB	Metabolism	19	22453500–22454000	Intron 13/29	4.43 (2.08)	11.92 (5.76)	0.91 (0.18)	0.19 (0)	0.038 (0.12)
PPP1R3B	Metabolism	16	60562000–60562500	Intron 1/1	4.08 (2.56)	1.00 (0)	0.49 (0)	6.85 (0)	0.026 (0.076)
SCD1	Metabolism	1	249463500–249464000	Intron 5/5	10.82 (4.11)	16.68 (5.85)	1.89 (1.58)	0.47 (0.40)	0.012 (0.037)

MeDIP and MRE values are given as average reads (standard deviation). Conservation denotes conservation score (standard deviation).
